# FairEmbo Concept for Postpartum Hemorrhage: Evaluation of the Efficacy of Suture Fragment Compared with Gelatin Sponge Torpedo Embolization in a Post-Gravid Swine Model

**DOI:** 10.3390/jpm13010124

**Published:** 2023-01-07

**Authors:** Amandine Banata Gang-Ny, Julien Panneau, Pauline Brige, Jean-François Hak, Paul Habert, Vincent Vidal, Farouk Tradi, Mathieu Di Bisceglie

**Affiliations:** 1 Department of Interventional Imaging, Assistance Publique Hopitaux de Marseille, 13005 Marseille, France; 2 LIIE, Aix Marseille University, Timone Campus, 13005 Marseille, France; 3 CERIMED, Aix Marseille University, Timone Campus, 13005 Marseille, France

**Keywords:** postpartum hemorrhage, uterine artery embolization (UAE), FairEmbo, suture, gelatin sponge

## Abstract

Background: Postpartum hemorrhage is the leading cause of maternal mortality in emerging countries. This study aims to evaluate the effectiveness and safety of uterine artery embolization (UAE) using suture fragment (FairEmbo concept) in a swine model. Methods: Seven female swine uteri were embolized. The left uterine artery was embolized with 1 cm fragments of absorbable suture (Optime^®^ 0), and with gelatin sponge torpedoes for the contralateral side for comparison. The embolization effectiveness and the time for arterial recanalization with digital subtraction angiography (DSA) controls at D0, D7, and M1, were evaluated. Follow-up protocol also included clinical monitoring and macroscopical analyses at M1. A Mann–Whitney test (significance at P 0.05) was used for statistics. Results: A technical success was obtained for the seven arteries on each side, with no off-target embolization. The procedure time (10 min versus 3.7 min) and number of fragments (13.8 versus 5.7) required for complete occlusion were significantly greater in the FairEmbo group. All arteries were repermeabilized at M1. No necrosis was macroscopically visible at harvest at M1. Conclusion: This experimental study suggests that UAE with SBM FairEmbo method is feasible, safe, and effective in comparison with gelatin sponge procedure.

## 1. Introduction

The risk of postpartum hemorrhage can occur during the first 24 h after delivery [1,2]. This classic complication is the leading cause of 100,000 annual deaths worldwide with 99% occurring in emerging countries [3,4].

For several years, uterine artery embolization (UAE) has demonstrated itself as an effective and safe alternative to hemostasis hysterectomies in treating severe postpartum hemorrhage [5,6,7,8,9]. This less morbid treatment also offers the option of subsequent pregnancies [7]. 

Gelatin sponge is the most common embolic agent used in UAE for postpartum hemorrhage for its resorbable properties [8]. In low- and middle-income countries this minimally invasive technique is not widespread for the management of postpartum hemorrhage, causing significant mortality, hysterectomy, and morbidity [9]. This can be attributed to costs, and also to the non-availability of various embolization agents, including the gelatin sponge. The lack of distribution of this type of medical product is a major cause of the shortage of embolization agents needed for this type of procedure.

The FairEmbo concept aims to make available a worldwide, effective, and safe embolization agent, either using absorbable or non-absorbable sutures. It has recently demonstrated that embolization with suture fragments is feasible and effective in short- and medium-term follow-up in porcine models [10,11]. Its clinical efficacy was also demonstrated by clinical case emergency embolization of a renal artery pseudoaneurysm in 2020 [12].

The technique using sutures as an embolization agent could be an alternative management for postpartum hemorrhage, by replacing gelatin sponges in those countries lacking medical material resources.

Our study aims to evaluate the feasibility of uterine artery embolization by using absorbable suture fragments and to compare the efficacy and risk of uterine necrosis caused by UAE with gelatin sponge embolization in porcine models.

## 2. Materials and Methods

### 2.1. Animal Model

This in vivo study of seven Pietrain pigs (40 ± 5 kg) was performed in a manner consistent with institutional and national guidelines for animal care and use. The study was approved by the Animal Ethics Committee. The swine’s uterus is bicornuate thus enabling a complete and separate evaluation of the right uterus’ vascularization by the right uterine artery, and the left uterus’ vascularization by the left uterine artery. A total of 14 uteri from seven nonpregnant adult female swine were used. Preparation of a post-gravid uterus model was carried out by daily intra-muscular administration of 6000 IU of chorionic gonadotropin for seven days before the embolization procedure. This procedure described in the literature has allowed to increase each uterine size and the diameter of the uterine arteries [13,14]. 

For each procedure, anesthesia was obtained with 2 mg/kg propofol and maintained with gaseous sevoflurane (2%) under mechanical respiration (Dräger Zeus, Dräger Inc., USA).

### 2.2. Embolization Agents

**Figure 1:**
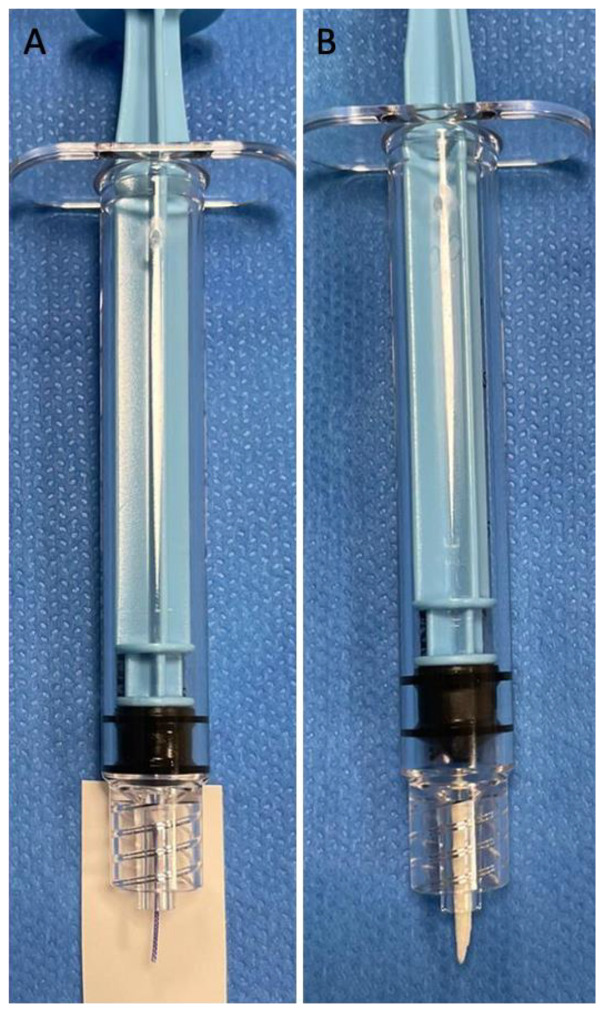
Comparison between a 1 cm long Optime 0 suture fragment (**A**) and a gelatin torpedo as usually shaped (**B**).

Absorbable polyglycolic acid (PGA) braided sutures Optime^®^ (Peters Surgical, Boulogne-Billancourt, France) were used. Fragments were obtained by a sterile cutting of the sutures during the embolization procedure with standard scissors with the objective of a one-centimeter-long fragment. The diameter of the suture was between 300 and 400 (size 0 USP, United States Pharmacopeia). The suture fragments were compared with an absorbable gelatin sponge torpedo usually used in UAE procedures according to the current practice performed in humans (Figure 1). The torpedoes were calibrated to approximately 1 cm as is typical for this type of procedure. The resorbable gelatin was derived from purified porcine gelatin (Curaspon^®^, Curamedical, Assendelft, The Netherlands) [15]. The left-sided uterus was embolized with centimetric suture fragments, and a comparative embolization with absorbable gelatin sponge was performed in the right-sided uterine artery.

### 2.3. Embolization Procedure

A digital subtraction angiography (DSA) system (Fluorostar, General Electric Medical System, Chicago, IL, USA) was used for the radiological procedures. Percutaneous access was performed by left carotid arterial puncture with the placement of a 5 French (F) vascular introducer by the Seldinger method. Catheterization of arterial vascular targets was performed by an experienced interventional radiologist using a Vertebral 4F catheter (Merit). Pre-embolization angiograms were performed with Visipaque (320 mg I/mL, GE-30%) to ensure correct positioning of the catheter in the target uterine artery before embolization. Embolization was performed either by suture fragments or by resorbable gelatin according to the side. All seven swine underwent uterine angiography controls to assess uterine artery occlusion immediately after the procedure and recanalization at 7 and 30 days after embolization to evaluate the recanalization time.

### 2.4. Technical Success and Evaluation of Secondary Outcomes

Technical success was defined as occlusion of the target artery without residual opacification. This was illustrated by a stagnation of the iodinated contrast medium in the uterine artery above the embolization site and an absence of opacification of the distal branches in the lower target territory. These parameters were confirmed on the DSA control performed five minutes after this end point was achieved. Secondary short-term endpoints were safety (non-targeted embolization), the embolization procedure time (delay between the first DSA acquisition before the initiation of embolization and the final one confirming technical success), and the number of fragments or torpedoes fragments injected.

The recanalization time by angiographic controls at day seven and one month after embolization was estimated. Swine UAE with temporary embolic agents is compatible with survival, and the animals could be followed for one month until verification of satisfactory uterine artery recanalization. Uterine artery recanalization was defined by a partial or total visualization of the uterine artery. During this follow-up, the swine underwent a daily clinical evaluation to look for signs of infection or inflammation (anorexia, signs of malnutrition, and abnormal behavior).

After angiographic control at one month, the animals were euthanized, and their uteri were removed. The uteri were fixed at 5.0% in neutral buffered formalin and photographed both front and back. Dark areas were considered necrotic.

### 2.5. Statistics

The parameters studied were collected and analyzed using Excel 2021 software (Microsoft, Redmond, WA, USA). A Mann–Whitney test was used to test significance, and a *p* value < 0.05 was considered significant for the embolization time and the number of fragments/torpedoes used for effective embolization.

## 3. Results

### 3.1. DSA Parameters, Technical Success

The selective uterine artery embolization procedure was possible in all seven pigs for a total of 14 embolizations (seven right and seven left). Embolization by suture was feasible for all animals and without technical failure or procedural complications.

**Figure 2:**
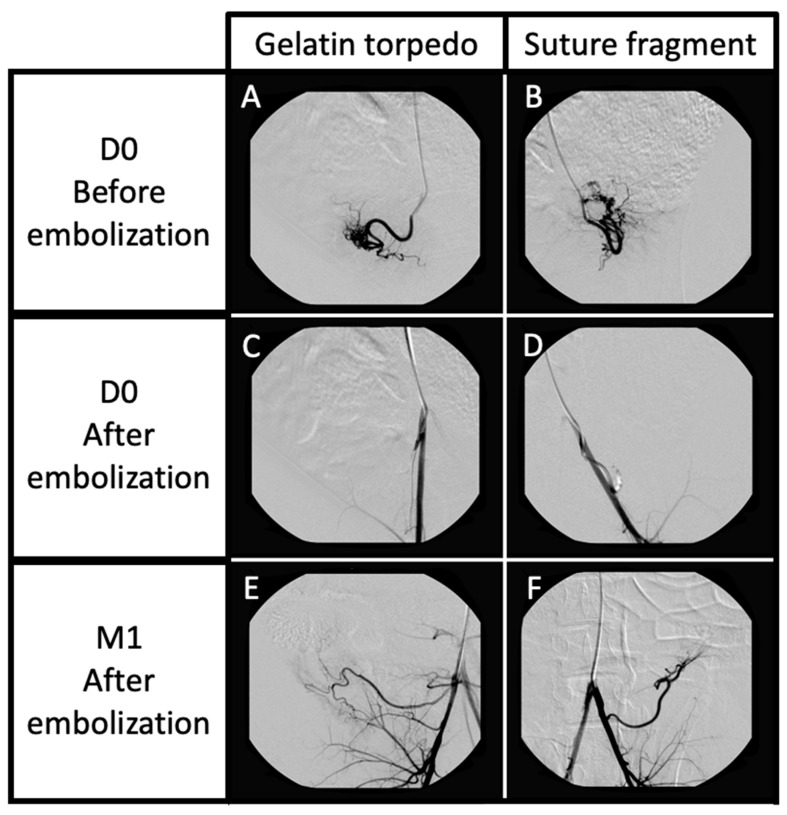
Angiography controls before uterine embolization (**A**,**B**), just after arterial embolization (right side by gelatin torpedo (**C**), left side by suture fragment (**D**)), and at one month after embolization (**E**,**F**). The DSA controls showed effective embolization on both sides, and complete recanalization at 1 month after embolization.

Angiographic control at the end of the initial procedure showed effective embolization for each uterus side (flow occlusion and no opacification in the downstream arterial branches), whether for the gelatin or the one-centimeter fragment suture procedures (Figure 2, Table 1).

### 3.2. Short-Term Secondary Endpoints

Regarding the secondary endpoints, the mean embolization time by suture fragments was 10 min versus 3.7 min for the resorbable gelatin group (*p* = 0.028). A mean of 13.8 suture fragments was required to achieve effective complete occlusion of the target uterine artery, compared with 5.7 torpedoes of absorbable gelatin (*p* = 0.029). 

There was no evidence of non-targeted embolization in either group.

### 3.3. Medium-Term Secondary Endpoints: ANGIOGRAPHIC Findings and Macroscopic Aspect

Three uterine arteries embolized with suture fragments were repermeabilized at D7 (43%) versus 7/7 for the gelatin group (100%). All uterine arteries were repermeabilized at M1 in both the suture and resorbable gelatin groups (100%) (Table 2).

The daily clinical examination follow-up did not show any sign of significant deterioration of general condition.

**Figure 3:**
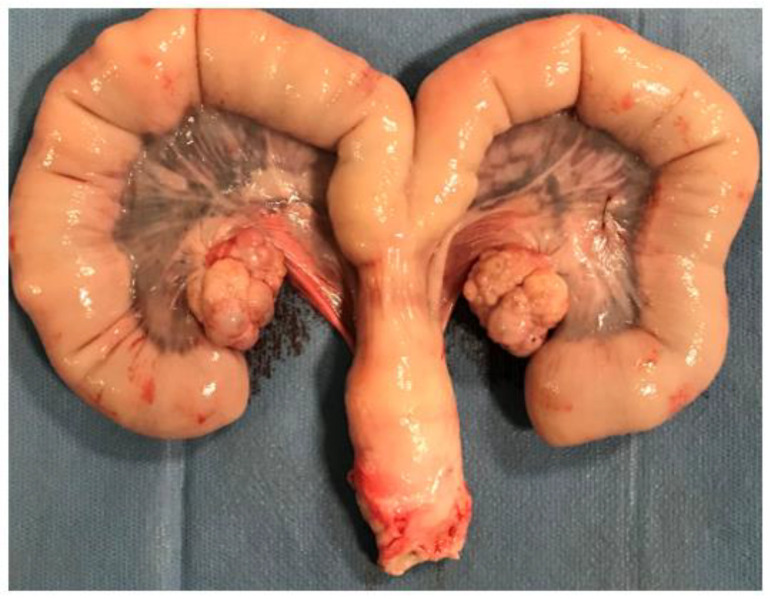
Uterus harvested. The right side was embolized with gelatin torpedoes, the left side was embolized with suture fragments. There was no difference in mucosal staining between the two sides, including no evidence of necrosis.

There was no evidence of right- nor left-sided uterine necrosis in any of the explanted uterine bodies. There was homogeneous and similar mucosal color between the two groups, with no significant difference in uterine trophicity (Figure 3).

## 4. Discussion

The feasibility of using suture fragments has been demonstrated in previous preclinical studies and through a clinical case reported in 2020 [10,11,12]. This study demonstrated the feasibility and efficacy of suture embolization through a new and previously untested application: uterine artery embolization in a female porcine model in which the uterine arteries were stimulated by gonadotropin hormone administration.

Embolization with suture fragments in hypertrophied uterine arteries proved to be feasible and effective compared with the gelatin torpedoes. Immediate angiographic controls showed proximal uterine artery occlusion in both the suture and gelatin groups in a comparable manner. It took longer to achieve angiographic success of the embolization with the FairEmbo technique because of the need to inject more wire fragments than the gelatin torpedoes. This was probably a consequence of the diameter differences in these two embolizing agents and the ability of the gelatin sponge to expand within the artery lumen, thus allowing a more rapid occlusion of the lumen. In an emergency situation, such as postpartum hemorrhaging, this may be an important element to take into account. However, the time it took to obtain optimal embolization in the suture group remained satisfactory. In addition, it was subjectively as easy to inject a torpedo as it was a suture fragment through the catheter.

The safety of suture fragment embolization was demonstrated by the absence of non-targeted embolization seen during angiographic controls. From this point of view, the results obtained are equivalent to those obtained by gelatin sponge embolization and account for the safe aspect of this embolization technique as has already been described in previous preclinical studies [10].

The sutures used were chosen for their resorbability in biological media and are already commonly used in vascular and digestive surgery. The use of braided sutures was preferred to monofilaments because of their superior thrombogenic properties [16]. Their resorbability in the bloodstream is an essential condition for their application in postpartum hemorrhaging where arterial embolization aims to preserve uterine integrity to avoid chronic ischemia and to achieve future pregnancies [17,18,19,20]. There are no in vivo studies that determine the resorbability time of this type of wire within the bloodstream. There are also no clinical studies to determine the resorbability time of gelatin sponge in human condition. In our study, this amount of time was greater for resorbable sutures (seven days to one month for almost half of the animals) than for gelatin torpedoes (less than seven days). This in vivo resorption time is also variable depending on the suture that can be used. The use of a rapid resorbable suture (e.g., rapidly resorbable PGA) could decrease the resorption time of the transient embolization agent and approach the data obtained by resorbable gelatin. A study comparing two resorbable sutures, one with rapid resorption and the other with non-rapid resorption, could thus be performed. However, no consequences on uterine vitality were observed, and with no macroscopic sign of uterine necrosis. This is probably due to the more or less rapid resorbable nature of the two agents used. It can also be additionally attributed to the proximal nature of embolization by a fragment of suture within the target uterine artery, such as the resorbable gelatin torpedoes compared to the gelatin slurry sometimes used for emergency uterine embolization, which is more conducive to ischemia by distal embolization. It may also be that despite a bi-horned uterus with each side vascularized by its own artery, there must be vascular anastomoses with the contralateral side on the one hand and the other arteries in the vicinity (notably gonadal) on the other hand. At last, it should be remembered that the coagulation parameters of the pig are different from those of the human being and may influence the speed of resorption of the embolic agents [21,22,23]. 

These pre-clinical results are already published, studying the FairEmbo technique with absorbable suture fragments, and showed no signs of necrosis on histological analysis within the targeted organs, suggesting safety against uterine necrosis in this application. Absorbable sutures based on polyglycolic acid are already commonly used in clinical practice, particularly for vascular and cardiac sutures, because of their biodegradable, biocompatible properties, and are approved by the FDA. Furthermore, the products of the hydrolysis of polyglycolic acid do not constitute an in vitro growth medium for bacteria [24,25]. However, the histological inflammatory parameters of the embolized uterus in an infection-prone postpartum period (endometritis) have not been studied. This could be carried out in a specific histological and biological study of the behavior and reaction of the uterine tissue after embolization with sutures, in comparison with the tissue effects of gelatin torpedoes usually used. Finally, this embolization with sutures remains to be evaluated in clinical practice.

Postpartum hemorrhage most often affects the populations in disadvantaged countries which suffer a lack medical equipment. The FairEmbo concept aims to make available a worldwide, effective, and safe embolization agent—either an absorbable or non-absorbable suture thread depending on clinical indications. Our study, a continuation of the studies already carried out, targets a common and worrying application in emerging countries in which this embolization technique is predestined. The ability to perform uterine artery embolization effectively and safely in the context of postpartum hemorrhage could save many maternal lives. However, further clinical studies are needed to develop the technique.

## Figures and Tables

**Table 1:** Immediate DSA endpoints. * Wilcoxon–Mann–Whitney test.

	Suture	Gelatin	*p* *
Immediate effective embolization (n)	7/7 (100%)	7/7 (100%)	
Embolization time (min)	10 (4–17)	3.7 (1–6)	0.028
Numbers of fragments/torpedoes used (n)	13.8 (4–25)	5.7 (3–10)	0.029
Non-targeted embolization (n)	0/7 (0%)	0/7 (0%)	

**Table 2:** Medium-term embolization parameters: assessment of arterial recanalization at D7 and M1.

	Suture	Gelatin
Arterial recanalization at D7 (n)	3/7 (43%)	7/7 (100%)
Arterial recanalization at M1 (n)	7/7 (100%)	7/7 (100%)

## Data Availability

Not applicable.
